# Femoral and tibial attachment positions of the anterolateral ligament graft are associated with anterior instability after combined anterior cruciate ligament and anterolateral ligament reconstruction

**DOI:** 10.1186/s43019-026-00342-4

**Published:** 2026-07-27

**Authors:** Se-Han Jung, Min Jung, Kwangho Chung, Hyun-Soo Moon, Byeong-Hun Jeon, Jun-Young Lim, So-Heun Lee, Sung-Hwan Kim

**Affiliations:** 1 Department of Orthopaedic Surgery, Yonsei University College of Medicine, Seoul, Republic of Korea; 2 Department of Orthopaedic Surgery, Severance Hospital, Seoul, Republic of Korea; 3 Department of Orthopaedic Surgery, Yongin Severance Hospital, Seoul, Republic of Korea; 4 Department of Orthopaedic Surgery, Gangnam Severance Hospital, Seoul, Republic of Korea; 5 Department of Medical Device Engineering and Management, Yonsei University College of Medicine, Seoul, Republic of Korea

**Keywords:** Anterior cruciate ligament, Anterior cruciate ligament reconstruction, Anterolateral ligament, Anterolateral ligament reconstruction, Graft position

## Abstract

**Background:**

While numerous studies have examined the benefits of combining anterior cruciate ligament (ACL) and anterolateral ligament (ALL) reconstruction on clinical outcomes, detailed investigations into the relationship between reconstructed ALL femoral and tibial attachment positions and outcomes remain limited. The purpose of this study is to analyze the association between ALL graft attachment positions and clinical outcomes in patients undergoing combined ACL and ALL reconstruction.

**Methods:**

Patients who underwent combined ACL and ALL reconstruction between 2019 and 2023 were retrospectively reviewed. Postoperative computed tomography was performed and reconstructed three-dimensionally to quantify the femoral and tibial graft attachment positions of the ALL. Clinical outcomes were assessed at 1- and 2-year follow-ups using objective and subjective measures, including anterior–posterior (AP) and rotational instability, range of motion, and several patient-reported outcomes. The associations between three-dimensionally measured graft attachment positions and clinical outcomes were analyzed.

**Results:**

A total of 52 patients were included. At 1 year, a more proximal and posterior ALL femoral position correlated with reduced AP instability measures including KT-2000 and Lachman stress radiograph side-to-side difference (SSD) (*r* = −0.305 to −0.354) and lower likelihood of a positive Lachman test (*r* = −0.414 and −0.586). Posterior femoral position was associated with greater extension deficit (*r* = 0.281, *P* = 0.043). Regression analysis showed that femoral AP position was associated with KT-2000 SSD (*B* = −0.327, *P* = 0.011) and Lachman positivity (posterior OR 0.60, *P* = 0.005; proximal OR 0.77, *P* = 0.032). At 2 years, posterior femoral AP position remained associated with reduced anterior translation (*B* = −0.301, *P* = 0.012), while the tibial AP position was positively associated with Lachman SSD (*B* = 0.297, *P* < 0.001), indicating that a more anterior tibial position, represented by a lower AP ratio, was associated with lower Lachman SSD. ACL tunnel position showed no significant association with clinical outcomes. Significant range of motion (ROM) deficits occurred in 21.2% at 1 year but declined to 5.7% (extension) and 11.4% (flexion) at 2 years. Anatomical femoral positioning was associated with reduced anterior instability (Lachman SSD at 1 year, *P* = 0.032; at 2 years, *P* = 0.008) but was also associated with greater early ROM deficits, which resolved by 2 years.

**Conclusions:**

The femoral and tibial attachment positions of the ALL graft were associated with AP instability after combined ACL and ALL reconstruction. A more horizontal graft course was associated with reduced AP instability and mild, transient early ROM deficits.

**Supplementary Information:**

The online version contains supplementary material available at 10.1186/s43019-026-00342-4.

## Introduction

Anterior cruciate ligament (ACL) reconstruction is a widely performed surgical procedure that aims to restore knee stability and function following ACL injury [1, 2 ]. Despite advancements in surgical techniques, a subset of patients continues to experience residual instability, which may compromise functional outcomes and increase the risk of graft failure [3–5]. Recently, renewed attention has been given to the anterolateral ligament (ALL), a structure thought to play a role in controlling internal rotation of the tibia during the pivot shift [6, 7 ]. Biomechanical and clinical studies have suggested that combined ACL and ALL reconstruction may provide superior stability compared with isolated ACL reconstruction [6, 8 ].

While several studies have investigated the clinical benefit of combining ALL reconstruction to ACL reconstruction, reporting benefits such as reduced graft rupture rates and improved rotational instability [9–11], less is known about the influence of the femoral and tibial attachment positions of the reconstructed ALL graft on clinical outcomes. Biomechanical studies have reported that femoral and tibial attachment positions influence graft behavior [12, 13 ]; however, due to limited clinical studies, their impact on real-world clinical outcomes remains unclear, including knee joint stability and subjective patient reported outcomes (PROs), compared with nonanatomical placement.

Our study aimed to analyze the impact of the femoral and tibial attachment positions of the reconstructed ALL graft on clinical outcomes in patients who underwent concurrent ACL and ALL reconstruction. Using postoperative three-dimensional (3D) reconstructed computed tomography (CT) images, we quantified the exact positions of the ALL graft attachment points and assessed their correlation with objective and subjective outcome measures, including anterior stability, knee joint range of motion (ROM), and PROs. We hypothesized that anatomic femoral attachment position (proximal and posterior to lateral femoral epicondyle [LFE] [14]) and anterior tibial attachment position of the ALL would be associated with improved anteroposterior (AP) and rotational stability after the combined ACL and ALL reconstruction.

## Methods

### Study design and patient selection

This retrospective cohort study primarily included patients who underwent combined primary ACL and ALL reconstruction between 2019 and 2023 at a single institution by a single senior orthopedic surgeon. All patients underwent an immediate postoperative computed tomography (CT) scan with informed consent to evaluate the ACL tunnel position and ALL graft attachment site. Exclusion criteria were as follows: (1) patients who were lost to follow-up within 1 year; (2) patients who required secondary arthroscopic debridement due to postoperative septic arthritis. The indication for ACL reconstruction was primarily in patients with ACL rupture confirmed on preoperative MRI, who demonstrated either grade 2 or higher anterior instability (Lachman test > grade 2, Lachman stress radiograph side-to-side difference [SSD] > 5 mm, or KT-2000 arthrometer test SSD > 5 mm) or rotatory instability (pivot shift test > grade 2), or who had meniscal tears requiring surgical repair. The surgical indication for ALL reconstruction performed combined with primary ACL reconstruction was intraoperative confirmation of high-grade pivot shift test of grade 2 or higher. Other associated injuries or additional risk factors were not used as indications for ALL reconstruction. Institutional review board approval was obtained prior to data collection, and due to the retrospective nature of the study and the minimal risk involved, patient consent was waived by the institutional review board approval.

### Surgical technique

All patients underwent arthroscopic ACL reconstruction with concurrent ALL reconstruction by a single senior surgeon. Before ACL reconstruction, a comprehensive intraarticular assessment was performed, and necessary arthroscopic procedures such as meniscal repair were completed. For younger, high-demand patients, autografts, primarily hamstring autografts, were preferentially recommended, while in highly active patients desiring an earlier return to sport, bone–patellar tendon–bone autografts were selectively utilized. All patients were informed of the pros and cons of autografts and allografts, and final graft selection was based on patient preference. When allografts were used, tibialis anterior allografts served as the graft source. ACL reconstruction was performed using an inside-out femoral tunnel drilling technique through an accessory anteromedial portal. Femoral fixation was achieved using suspensory fixation (Endobutton CL; Smith & Nephew, Watford, UK), and tibial fixation was performed with a bioabsorbable interference screw (Smith & Nephew, Watford, UK), followed by supplementary post-tie fixation with a cancellous screw and washer. The surgical technique followed protocols described in previous studies [15, 16 ].

**Fig. 1:**
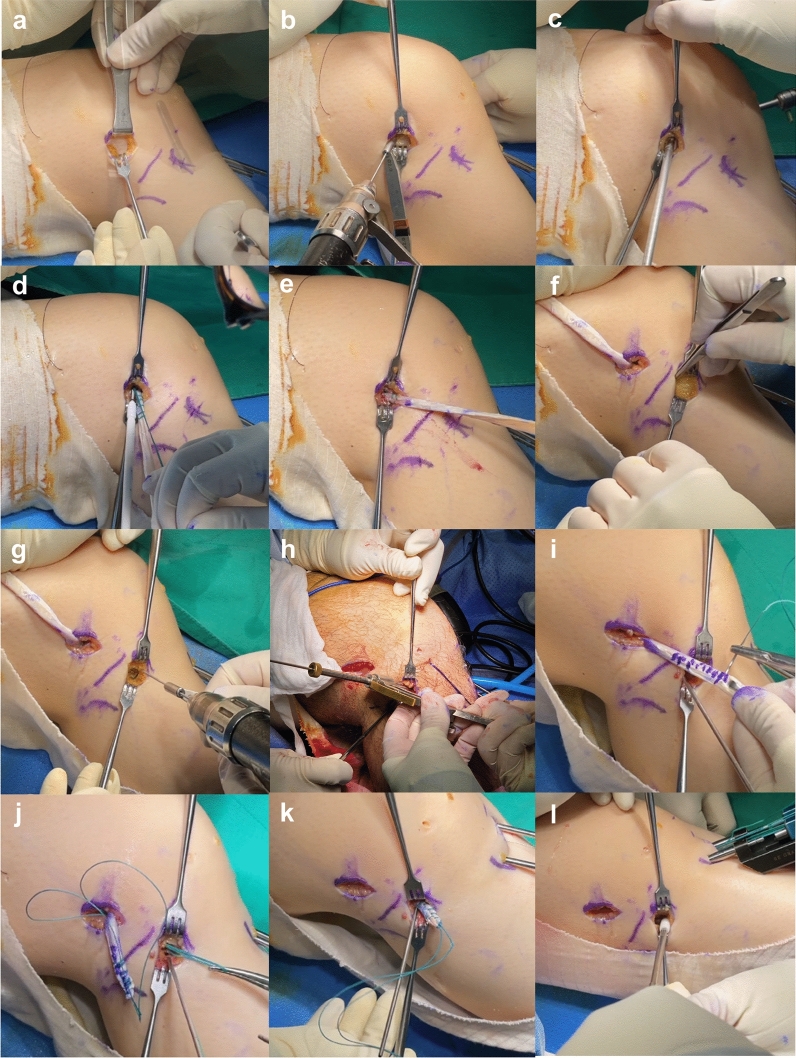
Single-arm anterolateral ligament (ALL) reconstruction procedure. **a** A longitudinal incision is made over the lateral femoral epicondyle to expose the iliotibial band, which is then incised to reveal the lateral epicondylar area. **b** A guide pin is inserted into the posterior–proximal aspect of the lateral femoral epicondyle to create a closed socket for suture anchor fixation (5.5-mm SwiveLock® anchor). The guide pin is directed distally to avoid collision with the anterior cruciate ligament (ACL) femoral tunnel. **c** A 6-mm reamer is used to create the femoral socket over the guide pin. To prevent intraarticular penetration, reaming is limited to approximately 25–30 mm. **d**, **e** The femoral side of the ALL graft is fixed within the prepared socket using a suture anchor. **f** A vertical incision is made posterior to Gerdy’s tubercle to expose and identify the ALL tibial attachment site. **g** A guide pin is inserted at the midpoint between Gerdy’s tubercle and the fibular head to identify the tibial attachment site. **h** When inserting the guide pin through to the opposite (medial) tibial cortex, an ACL tibial guide can be used to accurately target the desired position and avoid overlap with the ACL tibial tunnel. **i** The graft is prepared with approximately 30 mm left beyond the distance from the femoral attachment to the inserted tibial guide pin, and the distal 30 mm is whipstitched. **j** The graft is passed deep to the iliotibial band through the extraarticular tissues, from the femoral to the tibial incision. **k** The whipstitched sutures are passed through the tibial tunnel using the guide pin and drawn into position by gentle traction. **l** With approximately 20 N of gentle traction applied, the graft is fixed in the tibial tunnel using a bioabsorbable interference screw at approximately 10–15° of knee flexion

The ALL was reconstructed using either a single-arm or an inverted V-shaped double-arm technique, with the majority of cases utilizing the single-arm technique. For single-arm reconstructions, tibialis anterior allografts were used. The graft was trimmed to fit a 5-mm diameter slot-sizing block. One end of the graft was whipstitched approximately 3 cm in length using No. 2 Ethibond sutures. For the femoral fixation, a longitudinal skin incision (20–30 mm) was made over the LFE, which was identified by manual palpation (Fig. 1a). The iliotibial band was split longitudinally, and the femoral attachment point was set proximal and posterior to the identified LFE, following established anatomical references [14, 17 ]. Soft tissue dissection was minimized to maintain a minimally invasive approach. A guide pin was inserted at the designated femoral attachment site, directed 40° distally to avoid tunnel collision with the ACL femoral tunnel, as recommended in a prior study (Fig. 1b). [18] A closed-socket femoral tunnel (6 mm in diameter) was created (Fig. 1c), and the whipstitched end of the graft was fixed using a 5.5-mm SwiveLock® anchor (Arthrex, Inc., Naples, FL, USA) (Fig. 1d, e). On the tibial side, the Gerdy’s tubercle and fibular head were identified, and a vertical incision (approximately 20 mm) was made just posterior to the Gerdy’s tubercle (Fig. 1f). The tibial attachment site was set midway between the Gerdy’s tubercle and fibular head, as per anatomical references [17]. A guide pin was inserted at this site, aiming toward the opposite cortex for tunnel creation (Fig. 1g, h) [19]. The graft was trimmed to approximately 30 mm longer than the measured distance between the femoral and tibial attachment sites and whipstitched at its distal 30 mm (Fig. 1i). The graft was then passed deep to the iliotibial band, passing through the extraarticular soft tissues from the femoral to the tibial incision (Fig. 1j). A 6-mm diameter tibial tunnel was reamed over the guide pin, and the graft was advanced through the tunnel using the whipstitch sutures for guidance and traction (Fig. 1k). The graft was fixed on the tibial side using a bioabsorbable interference screw while applying approximately 20 N of gentle traction, with the knee at approximately 10°–15° of flexion and in neutral rotation (Fig. 1l). The inverted V-shaped double-arm ALL reconstruction was performed as previously described [20]. For both single-arm and double-arm techniques, the same graft tension was applied, with graft fixed under approximately 20N of traction.

Postoperatively, patients were allowed to ambulate with crutches and tolerable weight-bearing while wearing a hinged brace. ROM exercises and quadriceps isometric strengthening began immediately. At 6 weeks, patients progressed to full weight-bearing and initiated closed kinetic chain exercises. Open kinetic chain exercises, jogging, and swimming were started at 6 months. Return to pivoting or jumping sports was permitted 9 months postoperatively.

### Postoperative imaging and 3D reconstruction

All patients underwent immediate postoperative CT scanning using a CT scanner Sensation 64 (Siemens Healthcare, Erlangen, Germany) with tube parameters set at 120–140 kVp and 86–140 mA. The slice thickness, field of view, and acquisition matrix were 0.6–1.0 mm, 195–333 mm, and 512 × 512 pixels, respectively. Digital Imaging and Communications in Medicine (DICOM) data from the CT scans were extracted from a Picture Archiving and Communication System (GE Medical Systems Information Technologies, Milwaukee, WI, USA) and imported into Mimics software (version 17; Materialize, Leuven, Belgium), for the reconstruction of a 3D volumetric model of the knee.

### Assessment of the position of the ALL attachments and ACL tunnel

**Fig. 2:**
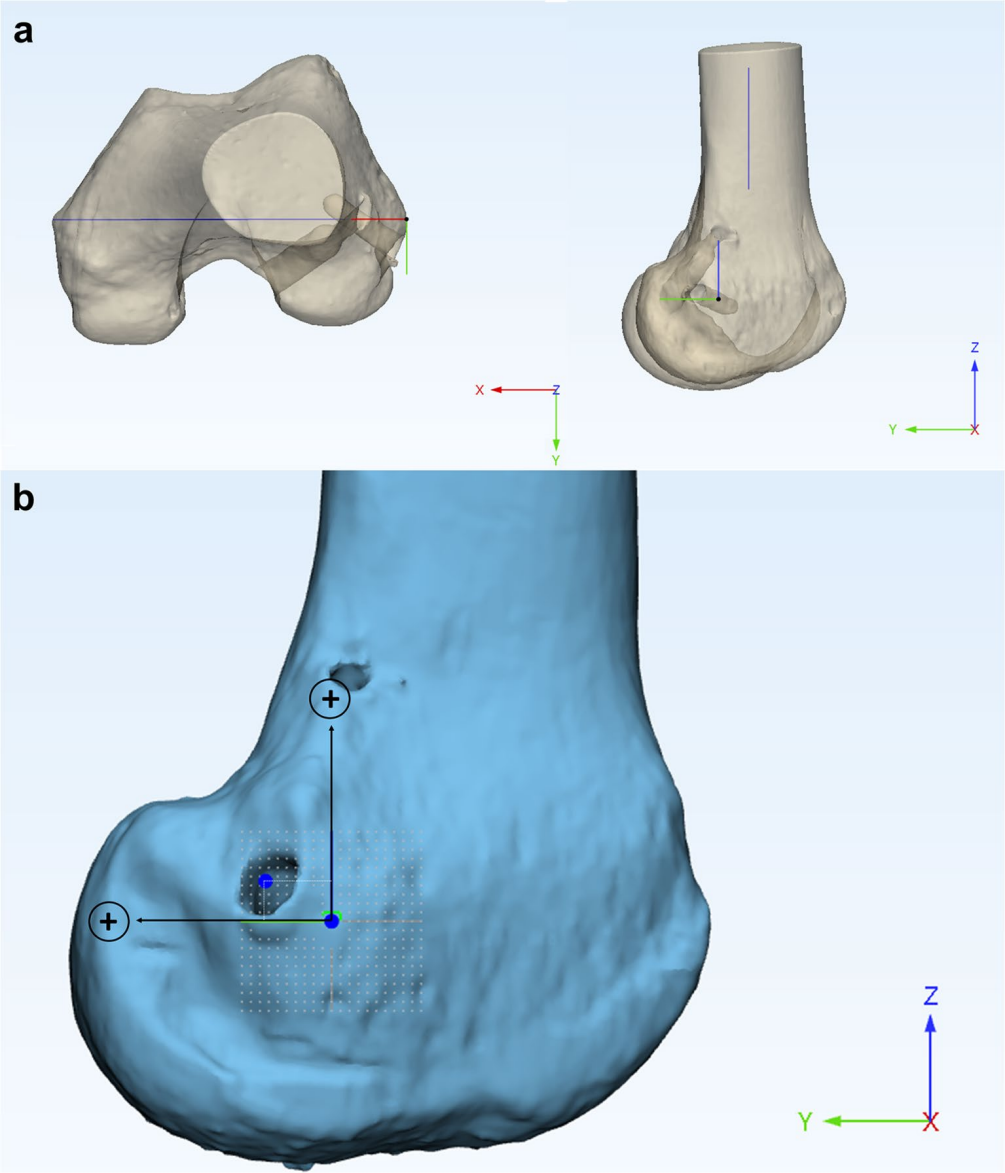
**a** Local coordinate setting of the femur. The *x*-axis was aligned with the femoral transepicondylar axis, and the *y*-axis was aligned parallel to the distal anatomical axis of the femur. **b** The lateral femoral epicondyle was set as the origin. The proximal direction was aligned with the *z*-axis, and the posterior direction with the *y*-axis. Proximal and posterior directions were designated as positive, respectively. The three-dimensional center of the ALL tunnel was then expressed as (, *z*) coordinates within this frame. Thereby, the *y*-coordinate indicates the anterior-to-posterior position of the ALL femoral attachment, and the *z*-coordinate indicates its distal-to-proximal position

For coordinate setting of the femoral model, the *x*-axis was aligned with the transepicondylar axis (TEA), with the zero point set at LFE. The *z*-axis was aligned with the distal femoral anatomical axis. The *y*-axis was set correspondingly (Fig. 2a). The femoral ALL attachment site, defined as the center of the ALL femoral tunnel orifice, was expressed using (*y*, *z*) coordinates, where positive values indicated positions posterior and proximal to the LFE, respectively (Fig. 2b). The *y*-coordinate represents the anterior-to-posterior (AP) position of the ALL femoral attachment, while the z-coordinate represents its distal-to-proximal (DP) position. Attachment sites were also categorized into four quadrants: Proximal Posterior, Proximal Anterior, Distal Posterior, and Distal Anterior, with proximal–posterior representing the anatomical attachment zone.

**Fig. 3:**
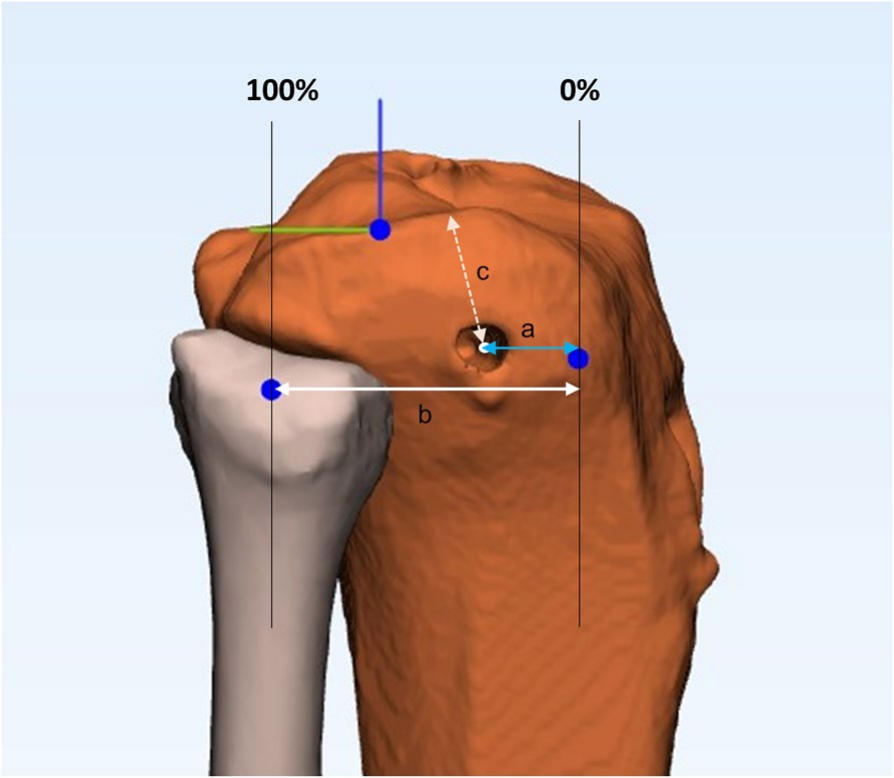
Assessment of the tibial anterolateral ligament (ALL) attachment. The anterior–posterior position of the ALL tibial attachment was calculated as a ratio (a/b × 100%): the distance from Gerdy’s tubercle to the ALL attachment site (a) divided by the total AP distance from Gerdy’s tubercle to the fibular head (b). The height of the ALL tibial attachment was assessed by measuring the vertical distance from the joint line to the attachment site, perpendicular to the joint line (c)

For ALL tibial attachment assessments, a new coordinate system was established for tibial model. The *x*-axis was aligned with the transtibial axis. The transtibial axis was defined by drawing two circles fitted to the medial and lateral tibial plateaus and connecting their centers with a straight line [21]. The *z*-axis was then aligned with the proximal tibial anatomical axis. The *y*-axis was again designated correspondingly. The AP position of the ALL tibial attachment was calculated as a ratio: the distance from Gerdy’s tubercle to the ALL attachment site divided by the total AP distance from Gerdy’s tubercle to the fibular head, based on the direct lateral (*yz*-plane) view (Fig. 3). The height of the ALL tibial attachment was assessed by measuring the vertical distance from the joint line to the attachment site, perpendicular to the joint line. The tibial attachment position was assessed only in patients who underwent single arm ALL reconstruction. Patients who underwent double-arm reconstruction were excluded from the tibial-side analysis because two distinct tibial attachment points were created, precluding the definition of a single representative tibial AP position using the present measurement method.

To evaluate the ACL tunnels, which can affect the surgical outcomes, the intraarticular tunnel orifice positions of the ACL femoral and tibial tunnels were measured on 3D CT using methods described in a previous study [15]. The position of the ACL femoral tunnel was expressed in terms of depth and height, while the position of the ACL tibial tunnel was expressed in terms of width and depth.

### Clinical outcome measures

Patients were assessed at 1-year and 2-year follow-up visits. Objective measures for anterior instability included side-to-side differences (SSD) in anterior translation using the KT-2000 arthrometer (MEDmetric Corp., San Diego, CA, USA) and Lachman stress radiographs. Physical examination, including the Lachman test and pivot shift test, was performed, and patient knee ROM was thoroughly assessed according to the International Knee Documentation Committee (IKDC) examination form [22]. Extension deficit and flexion deficit were expressed as the differences compared with the contralateral, nonoperated side. An extension deficit of 3° or more and a flexion deficit of 6° or more were defined as significant deficits, based on the downgrade criteria to Grade B in the IKDC examination form [22]. Subjective clinical outcomes were assessed using several patient-reported outcomes (PROs) including pain visual analogue scale (VAS), Lysholm knee score [23], IKDC subjective score [24], Knee injury and Osteoarthritis Outcome Score (KOOS) [25], and Tegner active scale [23].

### Statistical analysis

Values are presented as mean ± standard deviation for continuous variables and number (proportion, %) for categorical variables unless otherwise indicated. A *P*-value < 0.05 was considered statistically significant. Statistical analyses were performed using IBM SPSS Statistics for Windows, version 28.0 (IBM Corp., Armonk, NY, USA). Correlations between ALL attachment positions and clinical outcomes (Knee joint instability, physical examination, PROs) were analyzed using Pearson or Spearman correlation coefficients, depending on the results of normality testing with Shapiro–Wilk test. Correlations between ACL tunnel position and clinical outcomes, which could act as a confounding factor, were also analyzed. To further evaluate the associations identified in the correlation analyses, regression analyses were performed for instability measures that showed significant correlations with ALL attachment-position variables. The candidate attachment-position variables were ALL femoral DP position, ALL femoral AP position, ALL tibial AP position, and the distance from the joint line to the tibial attachment. For continuous instability outcomes, including KT-2000 SSD and Lachman stress radiograph SSD, multiple linear regression analyses were performed using the attachment-position variables that demonstrated significant correlations with each outcome in the preceding correlation analysis. For categorical instability outcomes, including Lachman test positivity and pivot-shift test positivity, logistic regression analyses were performed using the corresponding attachment-position variables that showed significant correlations. For multiple linear regression analyses, multicollinearity was assessed using variance inflation factors, and model assumptions were evaluated by visual inspection of residual plots. Group comparisons were conducted using *t*-tests or Mann–Whitney *U* tests depending on the normality testing (Shapiro–Wilk test) for continuous variables and chi-square tests or Fisher’s exact test for categorical variables. No formal adjustment for multiple comparisons was performed because the analyses were exploratory. ALL and ACL tunnel positions and Lachman stress radiograph SSD were evaluated by two evaluators with at least a 3-week interval to assess inter- and intra-observer reliability. Reliability was analyzed using intraclass correlation coefficients (ICC).

## Results

**Fig. 4:**
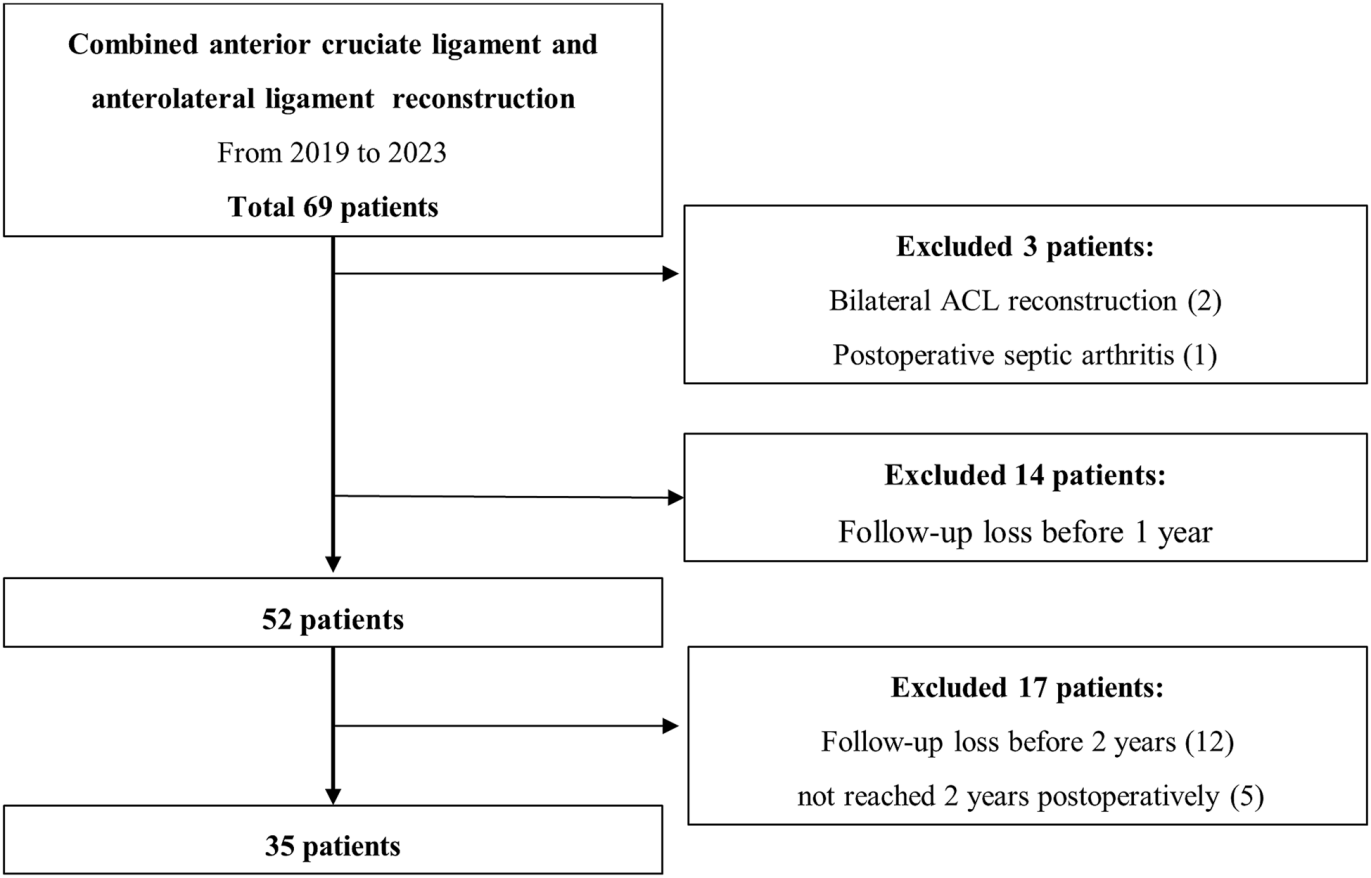
Patient flow diagram. ACL, anterior cruciate ligament

A total of 52 patients who underwent combined ACL and ALL reconstruction were included in the study. All patients had a minimum follow-up of 12 months, and 35 patients were followed for 24 months (Fig. 4). All 52 patients had complete clinical outcome data at the 1-year follow-up, and all 35 patients who completed the 2-year follow-up had complete clinical outcome data at that time point. No graft failure was observed during the follow-up period. Demographic characteristics and surgical profiles of the patients were described in Table 1.

**Table 1:** Demographic characteristics, surgical profiles, and assessment of ALL femoral/tibial attachment position

Variables (*n* = 52)	Values
Age, years	31.3 ± 12.9
Sex, male/female	41 (78.8)/11 (21.2)
Body mass index	24.8 ± 4.6
Affected side, right/left	28 (53.8)/24 (46.2)
ACL reconstruction	
Graft type, allogenous tibialis anterior graft / hamstring autograft / BPTB graft	20 (38.5)/28 (53.8)/4 (7.7)
Graft diameter, femoral side/tibial side, mm	8.4 ± 0.6/8.8 ± 0.6
Femoral tunnel orifice position, depth/height, %	28.3 ± 4.3/28.2 ± 6.8
Tibial tunnel orifice position, width/depth, %	44.6 ± 2.0/35.4 ± 4.7
Medial meniscus procedure	
Intact meniscus	29 (55.8)
Partial meniscectomy	3 (5.8)
Meniscal repair	20 (38.5)
Lateral meniscus procedure	
Intact meniscus	33 (63.5)
Partial meniscectomy	6 (11.5)
Meniscal repair	13 (25.0)
ALL reconstruction	
Single-arm/double-arm	43 (82.7)/9 (17.3)
Graft type (allograft/autograft)	40 (76.9)/12 (22.1)
Graft diameter, mm	5.3 ± 0.5
ALL Femoral attachment	
DP position, mm	4.4 ± 4.1
AP position, mm	3.9 ± 3.5
ALL Femoral attachment group	
Proximal posterior	40 (76.9)
Proximal anterior	6 (11.5)
Distal posterior	5 (9.6)
Distal anterior	1 (1.9)
ALL Tibial attachment	
AP position, %	33.0 ± 9.9
Distance from joint line, mm	14.2 ± 2.9
ALL Tibial attachment AP position	
Anterior one-third	24 (55.8)
Middle one-third	19 (44.2)
Posterior one-third	0 (0)

ALL, anterolateral ligament; ACL, anterior cruciate ligament; BPTB, bone-patellar tendon-bone; DP, distal to proximal; AP, anterior to posterior

### Clinical outcomes

All joint instability measures and PROs showed significant improvement at both 1-year and 2-year follow-ups compared with preoperative values (*P* < 0.001) (Table 2).

**Table 2:** Clinical outcomes—knee instability and patient-reported outcomes (preoperative, postoperative 1-year, and 2-year follow-up)

	Preoperative	Postoperative 1-year*	Postoperative 2-year*
KT-2000 SSD	7.6 ± 2.5	1.4 ± 2.4	1.9 ± 2.4
Lachman Telos SSD	6.0 ± 3.8	2.1 ± 3.2	1.8 ± 3.0
Lachman test, grade 0/1/2/3	0/0/45/7	36/16/0/0	22/13/0/0
Pivot shift test	0/0/29/23	48/4/0/0	31/4/0/0
Flexion deficit	–	3.2 ± 5.4	2.1 ± 3.9
Extension deficit	–	1.2 ± 2.3	0.4 ± 1.2
Extension deficit > 3 degree	–	11 (21.2)	2 (5.7)
Flexion deficit > 6 degree	–	11 (21.2)	4 (11.4)
Pain VAS	24.7 ± 24.4	11.6 ± 11.5	11.9 ± 16.9
Lysholm knee score	68.8 ± 22.9	83.6 ± 13.2	85.6 ± 9.9
IKDC—Symptom	20.9 ± 6.9	25.4 ± 5.9	26.4 ± 6.1
IKDC—Sports	21.2 ± 9.3	30.1 ± 6.4	32.9 ± 4.6
IKDC—Function	3.8 ± 2.3	6.5 ± 2.2	6.9 ± 2.1
IKDC—Total	51.3 ± 16.9	70.3 ± 14.0	75.2 ± 12.9
KOOS—Pain	73.4 ± 17.6	86.5 ± 11.4	87.6 ± 12.3
KOOS—Symptom	67.1 ± 22.5	74.5 ± 14.4	76.6 ± 17.9
KOOS—ADL	78.1 ± 20.4	94.1 ± 9.1	93.4 ± 10.4
KOOS—Sports	37.4 ± 27.4	66.7 ± 21.6	69.2 ± 25.4
KOOS—QOL	41.1 ± 22.1	61.4 ± 18.7	63.2 ± 20.9
Preinjury Tegner score	5.9 ± 1.7	–	–
Tegner score	2.0 ± 1.4	3.4 ± 1.5	4.1 ± 1.8

Data were available for 52 patients at the 1-year follow-up and 35 patients at the 2-year follow-up for all reported outcomes. VAS, visual analog scale; IKDC, International Knee Documentation Committee subjective score; KOOS, Knee injury and Osteoarthritis Outcome Score

*All patient-reported outcomes showed statistically significant improvement compared with the preoperative baseline (*P* < .001)

### Correlation of clinical measures with ALL and ACL Tunnel Position (Table 3)

**Table 3:** Correlation between ALL/ACL femoral and tibial tunnel position and outcomes

ALL	ALL femoral tunnel DP position		ALL femoral tunnel AP position		Tibial tunnel AP position		Distance from joint line to tibial tunnel	
	*r*	*P*	*r*	*P*	*R*	*P*	*r*	*P*
1-Year follow-up								
KT-2000 SSD	−0.316	0.022*	− 0.329	0.017*	−0.119	0.447	0.184	0.238
Lachman Telos SSD	−0.354	0.010*	−0.305	0.028*	0.021	0.895	0.198	0.204
Lachman test	−0.414	0.002*	−0.586	< .001*	0.118	0.450	0.039	0.805
Pivot shift test	−0.188	0.183	−0.091	0.519	0.169	0.278	0.133	0.393
Extension deficit	−0.061	0.666	0.281	0.043*	−0.183	0.240	−0.067	0.672
Flexion deficit	−0.002	0.987	−0.001	0.994	−0.243	0.117	−0.030	0.848
2-Year follow-up								
KT-2000 SSD	−0.159	0.362	−0.483	0.003*	0.099	0.617	0.184	0.238
Lachman Telos SSD	−0.057	0.751	−0.367	0.033*	0.450	0.019*	0.198	0.204
Lachman test	−0.164	0.347	−0.527	0.001*	0.415	0.028*	0.039	0.805
Pivot shift test	−0.196	0.260	0.062	0.722	0.107	0.587	0.133	0.393
Extension deficit	−0.151	0.386	0.137	0.432	−0.327	0.089	−0.067	0.672
Flexion deficit	0.207	0.233	−0.058	0.740	−0.208	0.289	−0.001	0.994

ACL	ACL femoral tunnel depth		ACL femoral tunnel height		ACL tibial tunnel width		ACL tibial tunnel depth	
	*r*	*P*	*r*	*P*	*r*	*P*	*r*	*P*
1-Year follow-up								
KT-2000 SSD	0.147	0.299	0.154	0.275	0.135	0.339	−0.015	0.918
Lachman Telos SSD	0.143	0.312	0.209	0.137	0.044	0.755	−0.029	0.841
Lachman test	0.082	0.564	0.193	0.170	0.047	0.740	−0.006	0.969
Pivot shift test	−0.079	0.576	−0.043	0.761	−0.142	0.316	−0.070	0.623
Extension deficit	−0.058	0.683	−0.121	0.394	−0.181	0.200	0.084	0.555
Flexion deficit	0.148	0.294	.017	0.903	0.130	0.360	0.074	0.601
2-Year follow-up								
KT-2000 SSD	0.105	0.459	0.104	0.552	0.112	0.521	−0.130	0.456
Lachman Telos SSD	−0.082	0.643	0.277	0.113	−0.205	0.245	−0.133	0.453
Lachman test	0.073	0.676	0.319	0.062	0.009	0.960	−0.111	0.525
Pivot shift test	−0.098	0.576	−0.125	0.476	−0.107	0.542	−0.031	0.859
Extension deficit	−0.067	0.703	−0.206	0.234	−0.171	0.327	−0.065	0.710
Flexion deficit	0.237	0.171	0.173	0.320	−0.014	0.936	−0.025	0.885

ALL, anterolateral ligament; ACL, anterior cruciate ligament; DP, distal to proximal; AP, anterior to posterior; SSD, side-to-side difference

*r* refers to the correlation coefficient value derived from the Spearman test. * Statistically significant

At 1 year postoperatively, correlation analysis revealed that a more proximal and posterior ALL femoral tunnel position was associated with reduced anterior translation as measured by KT-2000 arthrometer and Lachman stress radiograph SSD (*r* = −0.305 to −0.354). Similarly, the likelihood of a positive Lachman test decreased with a more proximal and posterior femoral attachment (*r* = −0.414 and −0.586). The AP position of the ALL femoral attachment showed a weak positive correlation with extension deficit (*r* = 0.281) indicating that a more posterior position was associated with increased extension deficit. In multiple linear regression analysis, the 1-year KT-2000 SSD was significantly influenced by the femoral AP position of the ALL tunnel (*R* = 0.498, *B* = −0.327, *P* = 0.011). Logistic regression analysis demonstrated that both the AP and DP positions of the ALL femoral tunnel significantly affected Lachman test positivity. Specifically, for each 1 mm that the ALL femoral tunnel was positioned more posteriorly or proximally, the likelihood of a positive Lachman test decreased (posterior position: odds ratio [OR], 0.60; 95% confidence interval [CI], 0.42–0.85; *P* = 0.005; proximal position: OR, 0.77; 95% CI 0.60–0.98; *P* = 0.032).

At 2 years postoperatively, the femoral AP position of the ALL tunnel remained significantly correlated with anterior tibial translation. Additionally, the tibial AP position of the ALL tunnel showed a correlation with Lachman stress radiograph SSD (*r* = 0.450) and Lachman test positivity (*r* = 0.415). In multiple linear regression, a more posterior femoral AP position was associated with decreased KT-2000 SSD (*R* = 0.479, *B* = −0.301, *P* = 0.012). Likewise, a more anterior tibial AP position was associated with decreased Lachman stress radiograph SSD (*R* = 0.757, *B* = 0.297, *P* < 0.001). Logistic regression demonstrated that the femoral AP position of the ALL tunnel significantly influenced the likelihood of a positive Lachman test (*P* = 0.036, OR 0.737; 95% CI 0.553–0.980). Detailed results of the regression analyses are provided in Supplementary Table 1–4.

In this cohort, ACL tunnel position did not significantly influence clinical parameters, including anterior tibial translation. The reliability of ACL femoral/tibial tunnel position measurements showed intra-observer ICC values of 0.916–0.969 and inter-observer values of 0.909–0.964. For ALL tunnel position assessment, intra-observer reliability ranged from 0.950 to 0.988, and inter-observer reliability from 0.943 to 0.982. The intra- and inter-observer reliability for Lachman Telos radiograph SSD measurements were 0.974 and 0.967, respectively.

### Extension and flexion deficit

Posterior femoral attachment positioning was correlated with the degree of extension deficit at 1 year (*r* = 0.281, *P* = 0.043). The incidence of significant extension was 21.2% (11/52) and that of significant flexion deficit was also 21.2% (11/52) at 1 year postoperatively, but decreased to 5.7% (2/35) and 11.4% (4/35), respectively, at 2 years, indicating gradual resolution over time.

### Subgroup analysis depending on ALL femoral and tibial tunnel position (femur: anatomical versus nonanatomical, tibia, anterior one-third versus middle one-third)

**Fig. 5:**
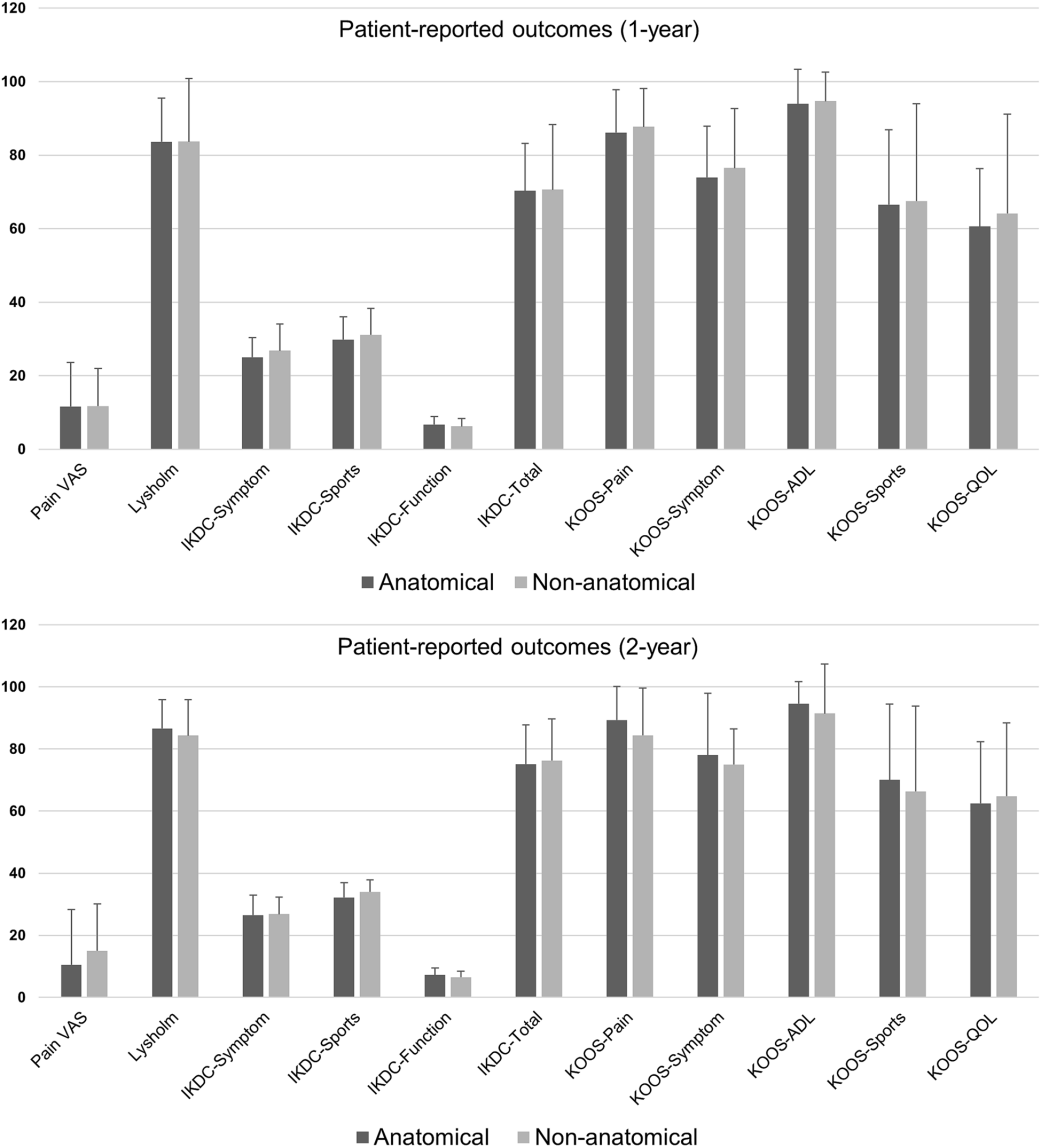
Patient-reported outcomes comparison between the anatomical group and nonanatomical group (anterolateral femoral attachment position). No significant differences were observed between the groups. VAS, visual analogue scale; IKDC, international knee documentation committee; KOOS, Knee injury and Osteoarthritis Outcome Score; ADL, activity of daily living; QOL, quality of life

When ALL femoral tunnel sites were categorized into anatomical (proximal posterior quadrant) versus nonanatomical positions, the anatomical group demonstrated significantly lower anterior instability on Lachman stress radiographs at both 1-year and 2-year follow-ups (Table 4). However, the 2-year subgroup comparison should be interpreted with caution because the nonanatomical group included only eight patients. The anatomical group demonstrated significantly greater extension and flexion deficits at 1 year postoperatively. However, at 2 years, no significant differences in extension or flexion deficits were observed between the two groups. There were no statistically significant differences between the two groups in PROs at both 1-year and 2-year follow-ups (Fig. 5). When patients were divided into anterior one-third and middle one-third groups on the basis of the tibial tunnel position, no significant differences were observed in anterior instability or ROM deficits at both 1 year and 2 years postoperatively (Table 4).

**Table 4:** Comparison of clinical outcome measures according to femoral and tibial ALL tunnel position

Femoral ALL tunnel position			
1-year follow-up	Anatomical (*n* = 40)	Nonanatomical (*n* = 12)	*P*-value
KT-2000 SSD	1.0 ± 2.4	2.5 ± 1.8	0.099^a^
Lachman Telos SSD	1.8 ± 3.2	3.4 ± 2.4	0.032^a^*
Lachman test, G0/1/2/3	12/9/0/0	5/7/0/0	0.031^b^*
Pivot shift test, G0/1/2/3	37/3/0/0	11/1/0/0	1.000^b^
Extension deficit	1.6 ± 2.6	0 ± 0	0.019^a^*
Flexion deficit	4.0 ± 5.8	0.8 ± 2.9	0.049^a^*

Femoral ALL tunnel position			
2-year follow-up	Anatomical (*n* = 27)	Nonanatomical (*n* = 8)	*P*-value
KT-2000 SSD	1.6 ± 2.4	3.0 ± 2.2	0.236^a^
Lachman Telos SSD	1.1 ± 2.9	3.9 ± 2.1	0.008^a^*
Lachman test, G0/1/2/3	19/8/0/0	3/5/0/0	0.116^b^
Pivot shift test, G0/1/2/3	23/4/0/0	8/0/0/0	0.553^b^
Extension deficit	0.6 ± 1.4	0 ± 0	0.451^a^
Flexion deficit	2.4 ± 4.1	1.3 ± 3.5	0.451^a^

Tibial ALL tibial position			
1-year follow-up	Anterior 1/3 (*n* = 24)	Middle 1/3 (*n* = 19)	*P*-value
KT-2000 SSD	1.3 ± 1.8	1.0 ± 3.0	0.903^a^
Lachman Telos SSD	1.7 ± 2.2	2.3 ± 4.4	0.714^a^
Lachman test, G0/1/2/3	19/5/0/0	11/8/0/0	0.131^c^
Pivot shift test, G0/1/2/3	24/0/0/0	17/2/0/0	0.189^b^
Extension deficit	1.4 ± 2.2	1.3 ± 2.8	0.416^a^
Flexion deficit	5.5 ± 6.8	1.6 ± 2.9	0.060^a^

Tibial ALL tibial position			
2-year follow-up	Anterior 1/3 (*n* = 16)	Middle 1/3 (*n* = 12)	*P*-value
KT-2000 SSD	1.4 ± 2.3	2.3 ± 2.4	0.189^a^
Lachman Telos SSD	0.7 ± 3.1	2.9 ± 3.1	0.074^a^
Lachman test, G0/1/2/3	13/3/0/0	5/7/0/0	0.050^b^
Pivot shift test, G0/1/2/3	14/2/0/0	11/1/0/0	1.000^b^
Extension deficit	0.8 ± 1.6	0 ± 0	0.280^a^
Flexion deficit	4.2 ± 5.0	0.7 ± 1.6	0.074^a^

Tibial-side analyses included only patients who underwent single-arm ALL reconstruction. ALL, anterolateral ligament; SSD, side-to-side difference. ^a^Mann–Whitney *U* test; ^b^Fisher’s exact test; ^c^chi-square test; *Statistical significance

### Additional subgroup and attrition analyses

No significant differences were observed in knee joint instability measures (including the Lachman test, pivot-shift test, KT-2000 SSD, and Lachman stress radiograph SSD) according to ACL graft type, ALL graft type, or ALL tibial fixation method at both 1- and 2-year follow-ups. In addition, no significant differences in baseline characteristics were observed between patients who completed the 2-year follow-up and those who were lost to follow-up. However, patients who were lost to follow-up demonstrated significantly better AP stability at 1 year, as indicated by a lower KT-2000 SSD, than those who completed the 2-year follow-up (0.4 ± 2.6 mm versus 1.8 ± 2.2 mm, *P* = 0.042).

## Discussion

**Fig. 6:**
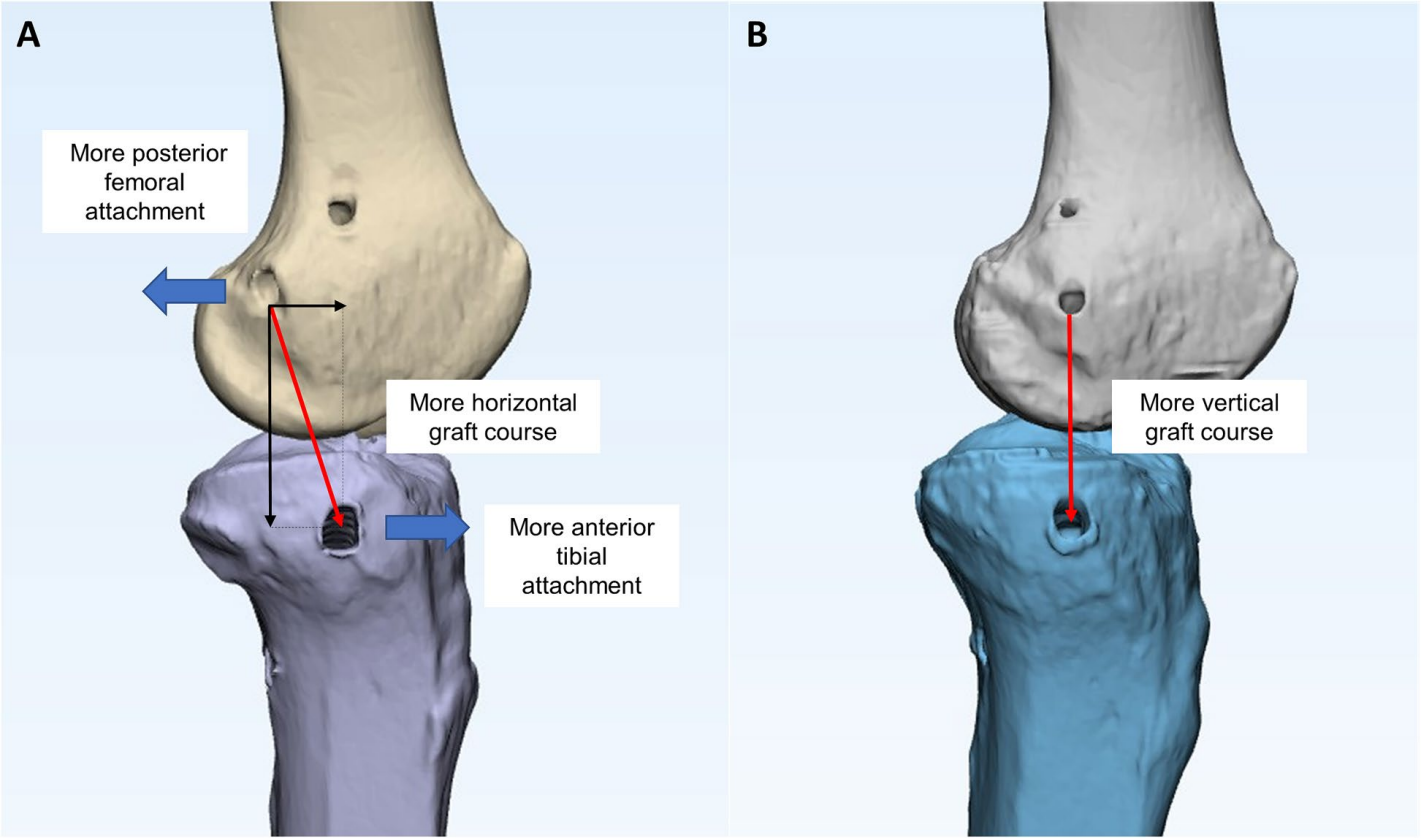
Schematic illustration of graft course orientation. **A** A more posterior femoral attachment combined with a more anterior tibial attachment creates a relatively more horizontal graft course. **B** In contrast, a less posterior femoral attachment or less anterior tibial attachment creates a relatively more vertical graft course

This study investigated the clinical relevance of femoral and tibial attachment positions in ALL reconstruction performed concomitantly with ACL reconstruction. In this cohort, low-grade pivot shift was observed in only 7.6–11.4% of patients up to 2 years postoperatively, and no high-grade pivot shift was observed, suggesting effective reduction of rotational instability after ALL reconstruction. This finding contrasts with previous reports, in which up to 35% of patients demonstrated a positive pivot shift in isolated ACL reconstruction [15, 26, 27 ]. Furthermore, the primary focus of this study—the femoral and tibial attachment sites in ALL reconstruction—appeared to be associated with AP instability rather than rotational instability. Specifically, a more proximal and posterior femoral attachment was associated with reduced AP instability at 1 year. At 2 years, a more posterior femoral attachment and a more anterior tibial attachment were associated with reduced AP instability. This combination of attachment positions was considered to create a relatively more horizontal graft course in the sagittal plane (Fig. 6), which was also associated with mild early postoperative ROM deficits.

Lateral extraarticular procedures (LEAP), including ALL reconstruction and lateral extra-articular tenodesis (LET), have been increasingly performed with ACL reconstruction, and previous studies have reported their benefits in reducing pivot shift and graft rerupture rates [9, 10, 28–32 ]. However, the effect of LEAP on AP instability has been less consistently evaluated because of variability in measurement methods across studies. Moreover, the clinical influence of femoral and tibial attachment positions of the reconstructed ALL graft remains unclear. To date, only cadaveric studies have investigated the biomechanical effects of varying ALL attachment positions [12, 13 ]. Katakura et al. [12] conducted a cadaveric study to evaluate the biomechanical effects of various femoral attachment sites. They compared three femoral positions relative to the LFE: proximal–posterior (anatomical), far proximal–posterior, and distal–anterior. Their findings showed that ALL reconstruction performed at the anatomical femoral attachment site, defined as approximately 8 mm proximal and 4 mm posterior to the LFE, effectively reduced tibial acceleration during pivot-shift testing. The far proximal–posterior attachment site did not reduce pivot shift but showed a greater, although statistically insignificant, reduction in anterior tibial translation compared with the anatomical position. Xu et al. [13] analyzed the biomechanical effects of different tibial attachment sites and reported that knee laxity was restored across all locations. Their data showed that as the tibial attachment site moved more anteriorly, anterior tibial translation decreased further and that a more anterior tibial attachment resulted in increased graft force during knee flexion, suggesting a potential risk of over-constraint in flexion. Consistent with these biomechanical findings, our study showed that femoral and tibial AP attachment positions were associated with objective AP instability measures. In addition, although not statistically significant, patients with tibial attachments located in the anterior one-third tended to demonstrate greater flexion deficits than those with attachment positions in the middle one-third. These findings suggest that the biomechanical influence of ALL attachment position may be reflected in both AP stability and early ROM, although the results should be interpreted cautiously given the exploratory nature of the study. In this study, AP instability was assessed using both KT-2000 arthrometer measurements and Lachman stress radiographs. However, the results obtained from these two measures did not consistently show the same associations with ALL attachment position at each follow-up time point, despite their expected similar trends. This discrepancy may be partly explained by methodological differences between the two assessments. In the present study, anterior translation on KT-2000 arthrometer measurement was evaluated under a manual maximum anterior load. Although all measurements were performed by a single examiner, the manually applied load may have varied slightly between examinations. Similarly, Lachman stress radiography may also be influenced by technical factors, including the knee flexion angle and the positioning of the stress device during image acquisition. These factors may have contributed to the inconsistency between the two AP instability measures.

In the present study, the term “more horizontal graft course” refers to the trajectory inferred from a more posterior femoral attachment combined with a more anterior tibial attachment. This orientation was not directly quantified by measuring a graft angle but was inferred from the independently measured femoral and tibial attachment positions. Although most studies have reported that ALL reconstruction has a limited effect on anterior translation [33–35], the present findings suggest that a more horizontal graft course may be associated with reduced AP instability. This interpretation is partly supported by biomechanical comparisons between ALL reconstruction and LET. Geeslin et al. [6] reported that, with 20 N of graft fixation tension, LET tended to reduce tibial internal rotation and anterior translation more than ALL reconstruction during pivot shift simulation. Inderhaug et al. [8] similarly showed that ALL reconstruction had a lesser effect on reducing internal rotation and anterior translation across flexion angles compared with LET procedures. This difference has been attributed to the different tibial attachment site [33, 36 ]. In LET, the tibial attachment is located at Gerdy’s tubercle, resulting in a more horizontal graft trajectory than ALL reconstruction [33, 36 ]. Therefore, in the present study, a more posterior femoral attachment combined with a more anterior tibial attachment may have created a relatively more horizontal graft course, potentially generating a greater posteriorly directed force and contributing to reduced AP instability.

Despite the benefits of reduced AP and rotational instability, LEAPs may carry a risk of over-constraint [11, 37 ]. Previous biomechanical studies have reported that ALL reconstruction can restrict tibial internal rotation beyond the intact ACL state and that ALL graft length change during knee motion is influenced by femoral and tibial attachment positions [6, 37, 38 ]. In our study, the anatomical femoral attachment group demonstrated greater extension and flexion deficits at 1 year, and femoral attachment position was significantly correlated with extension deficit. This may be partly explained by previous findings showing that a posterior femoral attachment causes the graft to lengthen as the knee extends from approximately 20° of flexion to full extension [38]. Because graft fixation in our cohort was performed at slightly flexed knee status (10–15° flexion), a posterior femoral attachment may have increased graft tension near full extension, potentially contributing to the observed extension deficit. Tibial attachment position may similarly affect graft behavior during flexion. A more anterior tibial attachment has been associated with greater graft lengthening and increased graft force during progressive knee flexion [13, 38 ] and patients with tibial attachments in the anterior one-third showed a tendency toward greater flexion deficit in our study. However, these ROM deficits appeared to be mild and partially resolved by the 2-year follow-up, possibly reflecting progressive rehabilitation, graft adaptation, or biological remodeling over time [39, 40 ]. However, because graft tension, graft length change, and biological remodeling were not directly evaluated, these mechanisms remain hypothetical.

In ALL reconstruction, it is often challenging to consistently position the graft at the exact anatomic or intended site [41]. Nevertheless, the present findings suggest that attachment positions producing a more horizontal graft course may be associated with reduced postoperative AP instability after combined ACL and ALL reconstruction. This potential benefit should be balanced against the possibility of early ROM restriction. However, the clinical relevance of the ROM findings should be interpreted cautiously because the mean extension deficit was small and between-group differences in ROM were no longer significant at the 2-year follow-up. Therefore, these findings likely represent mild and transient early postoperative ROM differences rather than persistent clinically meaningful stiffness. Careful postoperative ROM monitoring and rehabilitation may be warranted in patients with attachment positions associated with a more horizontal graft course.

Despite the observed associations with objective AP instability and early ROM deficits, no significant differences in PROs were observed according to ALL attachment positions. This discrepancy may be explained by the relatively small absolute differences in laxity and ROM, which may not have been large enough to be perceived by patients or reflected in subjective outcome scores within the relatively short follow-up period. In addition, PROs after ACL reconstruction are influenced by multiple factors beyond residual laxity, including pain, muscle strength, meniscal status, activity level, rehabilitation, and patient expectations. Therefore, the present findings should be interpreted as associations with objective stability measures rather than evidence of superior patient-perceived outcomes. Although the observed correlations were generally weak to moderate, postoperative instability after ACL reconstruction is multifactorial, and a single attachment-position variable would not be expected to explain a large proportion of clinical variability. Therefore, these findings suggest that ALL attachment positions may contribute to objective AP stability, but their clinical meaningfulness should be interpreted cautiously. In particular, the observed differences did not translate into clear differences in PROs during the short-term follow-up period. Longer-term follow-up is needed to determine whether these objective stability differences are associated with clinically important outcomes such as recurrent instability, graft failure, or revision surgery [9].

The attrition analysis should also be considered when interpreting the 2-year findings. Although baseline characteristics were comparable between patients who completed the 2-year follow-up and those who were lost to follow-up, the latter group demonstrated significantly better AP stability at 1 year, as indicated by a lower KT-2000 SSD. Therefore, the patients available at 2 years may have represented a cohort with relatively less favorable AP stability compared with the original study population. This potential selection bias may have amplified the apparent associations between ALL graft attachment position and AP instability observed at 2 years. Nevertheless, because the reasons for loss to follow-up were not systematically documented, it cannot be determined whether the observed difference reflects better clinical recovery among patients who did not return or other unmeasured factors. Accordingly, the 2-year findings should be interpreted cautiously.

This study has several limitations. First, its retrospective exploratory design and relatively small sample size introduce the possibility of selection bias and limit the generalizability of the findings. An a priori sample size calculation was not performed, and the limited number of patients, particularly at the 2-year follow-up and in the nonanatomical femoral attachment group, may have reduced the statistical power of subgroup and regression analyses. Specifically, the significant 2-year subgroup difference between the anatomical and nonanatomical femoral attachment groups should be interpreted cautiously because the nonanatomical group included only eight patients at the 2-year follow-up. In addition, as noted above, the loss to follow-up at the 2-year assessment may have introduced attrition bias. Second, although a rigorous 3D measurement method was developed and applied, there is currently no universally accepted standardized protocol for assessing ALL attachment positions. Therefore, some measurement error may remain, despite the potential advantages of 3D assessment over conventional two-dimensional radiographic methods. Third, rotational instability was assessed subjectively using the pivot-shift test without an objective quantitative measurement, and examiner-dependent variability may therefore have influenced the reliability of this assessment. Furthermore, generalized ligamentous laxity and medial meniscus ramp lesions, which may affect postoperative instability, were not systematically evaluated or controlled for. Fourth, although no significant differences in instability outcomes were observed according to ACL graft type, its potential confounding effect cannot be completely excluded because of the limited sample size. Both single-arm and double-arm ALL reconstruction techniques were also included. Although the same graft tensioning and fixation protocol was applied, differences in graft configuration and biomechanical behavior may have introduced heterogeneity and influenced the clinical outcomes. Fifth, no formal correction for multiple comparisons was performed; therefore, the possibility of type I error cannot be excluded. Although the regression models were limited to predefined attachment-position variables to reduce overfitting, the limited sample size, especially at the 2-year follow-up, may have affected the stability of the regression estimates. Accordingly, the findings should be interpreted as exploratory associations rather than confirmatory results. Finally, the follow-up period was relatively short. Further long-term and multicenter studies with larger cohorts are required to validate these findings.

## Conclusions

The femoral and tibial attachment positions of the ALL graft were associated with AP instability after combined ACL and ALL reconstruction. A more horizontal graft course was associated with reduced AP instability and mild, transient early ROM deficits, although these exploratory findings did not translate into clear differences in PROs.

## Supplementary Information

Supplementary material 1.

## Data Availability

The data that support the findings of this study are available from the corresponding author upon reasonable request.

## Abbreviations

ACL: Anterior cruciate ligament
ALL: Anterolateral ligament
AP: Anterior–posterior
CI: Confidence interval
CT: Computed tomography
DICOM: Digital Imaging and Communications in Medicine
DP: Distal–proximal
ICC: Intraclass correlation coefficient
IKDC: International Knee Documentation Committee
KOOS: Knee injury and Osteoarthritis Outcome Score
LEAP: Lateral extra-articular procedure
LET: Lateral extra-articular tenodesis
LFE: Lateral femoral epicondyle
MRI: Magnetic resonance imaging
OR: Odds ratio
PROs: Patient-reported outcomes
ROM: Range of motion
SD: Standard deviation
SSD: Side-to-side difference
TEA: Transepicondylar axis
VAS: Visual analogue scale
