# Supplementary material for: Femoral and tibial attachment positions of the anterolateral ligament graft are associated with anterior instability after combined anterior cruciate ligament and anterolateral ligament reconstruction
Source: Knee Surg Relat Res. 2026 Jul 27;38:34. doi: 10.1186/s43019-026-00342-4 (PMC13410599; doi:10.1186/s43019-026-00342-4)
Supplement: Supplementary file 1 — Supplementary material 1. [file 43019_2026_342_MOESM1_ESM.docx]

**Supplementary table 1.** Multiple linear regression analysis of ALL femoral attachment position for 1-year KT-2000 side-to-side difference

| Dependent variable |  | Predictor variable | B | 95% CI for B | β | P-value | VIF |
| --- | --- | --- | --- | --- | --- | --- | --- |
| 1-year KT-2000 SSD |  | ALL femoral DP position, mm | 0.037 | −0.242 to 0.316 | 0.051 | .785 | 1.078 |
|  |  | ALL femoral AP position, mm | −0.327 | −0.570 to −0.084 | −0.510 | .011* | 1.078 |

Model summary: R = 0.498, R² = 0.248, model P = .033.

ALL, anterolateral ligament; AP, anterior-to-posterior; CI, confidence interval; DP, distal-to-proximal; SSD, side-to-side difference; VIF, variance inflation factor. * Statistically significant

**Supplementary Table 2.** Logistic regression analysis of ALL femoral attachment position for 1-year Lachman test positivity

| Dependent variable | Predictor variable | B | SE | Wald | OR | 95% CI for OR | P-value |
| --- | --- | --- | --- | --- | --- | --- | --- |
| 1-year Lachman test positivity | ALL femoral DP position, mm | −0.268 | 0.125 | 4.602 | 0.765 | 0.598 to 0.977 | .032* |
|  | ALL femoral AP position, mm | −0.519 | 0.183 | 8.044 | 0.595 | 0.416 to 0.852 | .005* |

Model summary: overall model χ² = 19.170, df = 2, P < .001.

Abbreviations: ALL, anterolateral ligament; AP, anterior-to-posterior; CI, confidence interval; DP, distal-to-proximal; OR, odds ratio; SE, standard error. * Statistically significant

**Supplementary Table 3.** Multiple linear regression analysis of ALL attachment position for 2-year KT-2000 side-to-side difference

| Dependent variable | Predictor variable | B | 95% CI for B | β | P-value |
| --- | --- | --- | --- | --- | --- |
| 2-year KT-2000 SSD | ALL femoral AP position, mm | −0.301 | −0.530 to −0.072 | −0.480 | .012* |
|  | ALL tibial AP position, % | −0.004 | −0.108 to 0.101 | −0.012 | .946 |

Model summary: R = 0.479, R² = 0.229, adjusted R² = 0.167, model P = .039.

Abbreviations: ALL, anterolateral ligament; AP, anterior-to-posterior; CI, confidence interval; SSD, side-to-side difference. * Statistically significant

**Supplementary Table 4.** Multiple linear regression analysis of ALL attachment position for 2-year Lachman stress radiograph SSD

| Dependent variable | Predictor variable | B | 95% CI for B | β | P-value | VIF |
| --- | --- | --- | --- | --- | --- | --- |
| 2-year Lachman stress radiograph SSD | ALL femoral AP position, mm | −0.136 | −0.417 to 0.145 | −0.163 | .323 | 1.023 |
|  | ALL tibial AP position, % | 0.297 | 0.157 to 0.437 | 0.715 | <.001* | 1.023 |

Model summary: R = 0.757, R² = 0.574, adjusted R² = 0.523, model P < .001.

Abbreviations: ALL, anterolateral ligament; AP, anterior-to-posterior; CI, confidence interval; SSD, side-to-side difference; VIF, variance inflation factor. * Statistically significant
